# Signatures of Environmental Genetic Adaptation Pinpoint Pathogens as the Main Selective Pressure through Human Evolution

**DOI:** 10.1371/journal.pgen.1002355

**Published:** 2011-11-03

**Authors:** Matteo Fumagalli, Manuela Sironi, Uberto Pozzoli, Anna Ferrer-Admettla, Linda Pattini, Rasmus Nielsen

**Affiliations:** 1 Scientific Institute IRCCS E. Medea, Bioinformatic Lab, Bosisio Parini, Italy; 2 Bioengineering Department, Politecnico di Milano, Milan, Italy; 3 Departments of Integrative Biology and Statistics, University of California Berkeley, Berkeley, California, United States of America; University of Washington, United States of America

## Abstract

Previous genome-wide scans of positive natural selection in humans have identified a number of non-neutrally evolving genes that play important roles in skin pigmentation, metabolism, or immune function. Recent studies have also shown that a genome-wide pattern of local adaptation can be detected by identifying correlations between patterns of allele frequencies and environmental variables. Despite these observations, the degree to which natural selection is primarily driven by adaptation to local environments, and the role of pathogens or other ecological factors as selective agents, is still under debate. To address this issue, we correlated the spatial allele frequency distribution of a large sample of SNPs from 55 distinct human populations to a set of environmental factors that describe local geographical features such as climate, diet regimes, and pathogen loads. In concordance with previous studies, we detected a significant enrichment of genic SNPs, and particularly non-synonymous SNPs associated with local adaptation. Furthermore, we show that the diversity of the local pathogenic environment is the predominant driver of local adaptation, and that climate, at least as measured here, only plays a relatively minor role. While background demography by far makes the strongest contribution in explaining the genetic variance among populations, we detected about 100 genes which show an unexpectedly strong correlation between allele frequencies and pathogenic environment, after correcting for demography. Conversely, for diet regimes and climatic conditions, no genes show a similar correlation between the environmental factor and allele frequencies. This result is validated using low-coverage sequencing data for multiple populations. Among the loci targeted by pathogen-driven selection, we found an enrichment of genes associated to autoimmune diseases, such as celiac disease, type 1 diabetes, and multiples sclerosis, which lends credence to the hypothesis that some susceptibility alleles for autoimmune diseases may be maintained in human population due to past selective processes.

## Author Summary

Adaptation to local environments is one of the most important factors shaping human genetic variation among different geographically distributed populations. Here we develop a statistical framework aimed at identifying signals of genetic adaptation. We correlate the spatial distribution of allele frequencies of a large sample of SNPs, genotyped in more than 50 populations distributed worldwide, to a set of environmental factors, describing local geographical features such as climate conditions, diet regimes, and pathogens load. Our results show an excess of putative functional variants for high levels of population differentiation, measured by the degree to which genetic variation correlates with a set of environmental variables. We demonstrate that selection on pathogens is the primary driver of local adaptation and affects the distribution of genetic variation at a large number of genes. Among the selected genes, we also identify an excess of genes associated with autoimmune diseases, such as celiac disease, type 1 diabetes, and multiples sclerosis.

## Introduction

Anatomically modern humans appeared in East Africa about 200 k years ago, spread out from sub-Saharan Africa approximately 100 k years ago, and subsequently colonized the rest of the world in a series of migratory events [1]. During this period humans encountered a wide range of different environmental conditions, which may have induced a number of genetic adaptations.

Recent evidence suggests that the observed phenotypic diversity among human population groups may to some extent be a product of local adaptive processes (e.g. reviewed in [2]), driven by regional variation in pathogen environment, diet, or climate [3]. For example, both genome-wide scans and studies on candidate loci identify genes under selection associated with skin pigmentation, presumably due to the different needs for skin protection in regions with different UV radiation intensity [4]-[8]. A number of genomic scans for loci under selection have been conducted in humans, using methods based on the distribution of SNP allele frequencies, [8]-[10], haplotype structure [4], [7], [11], [12], strength of population subdivision [13]-[15] or a combination of multiple measures [16]. These scans all attempt to identify the signature of a recent selective sweep (the effect of an advantageous mutation as it increases in frequency in the population). Most of these methods have reasonable power to detect a ‘hard sweep’, i.e. a sweep caused by a single new advantageous mutation affected by strong selection. However, they do not identify the underlying environmental factors (if any) that induced the selection acting on the target gene. Additionally, it has been recently suggested that most selection in humans may not be caused by hard sweeps, but rather by selection acting on standing variation in many genes (‘soft sweeps’) [17], [18]. Much evidence for selection may have been missed by focusing strongly on hard sweeps.

A promising alternative strategy for elucidating signatures of human local adaptation, especially when individual beneficial variants have a weak phenotypic effect, is to identify polymorphisms that strongly correlate in frequency with environmental variables [19]. Indeed, heat adaptation in human populations has been shown to correlate with latitude, precipitation and temperature [20]. Based on this observation Young and colleagues hypothesized that past adaptation to climate may be the main cause for the current widespread susceptibility to hypertension [20]. More recently, a scan for selection in candidate genes involved in metabolic disorders, suggested increases levels of positive selection in these disease pathways due to adaptation to local climatic conditions [21]. Similarly, signatures of adaptations to local dietary specializations have been observed [22].

Infectious diseases are one of the most important causes of mortality in human populations. Polymorphisms associated with response to infectious diseases are, therefore, likely targets of selection. Human genetic adaptation may to a large extent be driven by response to microbial, viral or parasite presence. Indeed, numerous studies have identified immune- and defense-related genes targeted by positive selection in the human genome [23]-[37] (reviewed in [38]).

By correlating population allele frequencies with local pathogen diversity, several studies have argued that pathogen-driven selection have been an important force in local adaptation in the MHC class I loci [39], blood group antigen genes [25], and interleukin genes and their receptors [27]. Also, genome-wide scans of adaptation to pathogens identified gene networks correlated with specific pathogen species such as viruses [40], protozoa [41] and helminthes (parasitic worms) [42].

Despite these observations, there is still great uncertainty about the relative importance of the role of pathogens and other ecological factors as selective agents in local adaptive process. Similarly, the degree to which adaptation to infectious agents or to other environmental factors has shaped the distribution of complex-disease alleles in humans is still under debate [24], [27], [38], [43]-[45].

The objective of this study is to identify signatures of human genetic adaptation to local environments, separating the contributions of different environmental factors such as climate, subsistence, and pathogenic environment. We show that the latter factor is the strongest driver of local adaptation, and identify specific pathways in the immune system, and specific disease susceptibility alleles, affected by selection related to the local pathogenic environment.

## Results

### Enrichment of genic SNPs for high values of prediction accuracy

We first identify possible signatures of human genetic adaptation to local environments via a statistical framework based on exploring correlations between population allele frequencies and environmental variables. Our major goal is to determine the relative contributions of different environmental predictors in driving local adaptation.

Its is often assumed that natural selection is more likely to act on genic rather than non-genic polymorphisms because the former are more likely to be of functional significance. In line with this assumption, previous studies have found a significant enrichment of genic polymorphisms among SNPs with high levels of population genetic differentiation [14], [46].

We verified these observations by correlating population allele frequencies of nearly 500k SNPs genotyped in 55 distinct human populations to a set of 14 environmental variables describing each geographic location (Table S1, Table S2, Table S3). By applying a Projection to Latent Structure multiple regression with an Uninformative Variable Elimination algorithm (UVE-PLS) we computed the prediction accuracy *Q* ^2^ for each SNP, and used this as a measure of how well the environmental variables predict distributions of allele frequencies. *Q* ^2^ serves here as a measure of genetic differentiation among populations, but instead of using geographic distances, or implicitly assuming an equal weight of all populations, population genetic differentiation is measured relative to the defined environmental variables. A high *Q* ^2^ value indicates that populations which are very different in terms of environmental variables also are very different in terms of allele frequencies.

**Figure 1:**
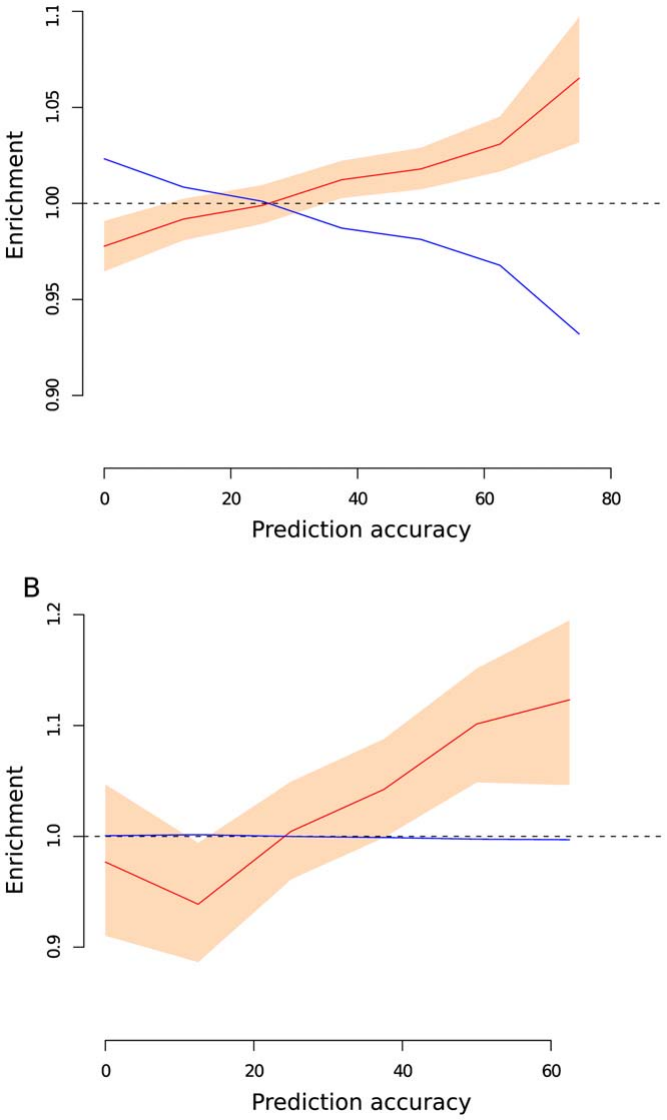
Enrichment of SNPs for different values of prediction accuracy. Enrichment of genic (red line, panel A) or non-synonymous (red line, panel B) vs. intergenic SNPs (blue line, both panel) for different values of prediction accuracy. Peach region denotes 90^th^ confidence interval computed with 1,000 bootstrap resamplings on overlapping blocks of 40 contiguous SNPs.

We examined the relative abundance of genic versus intergenic SNPs in the upper tail of the distribution of *Q* ^2^ values, as in the study by Coop *et al.* [46]. Significance and confidence intervals were determined using the Moving Block Bootstrap (MBB) estimates (see Materials and Methods). Notably, we found an enrichment of genic SNPs compared to non-genic SNPs for high values of prediction accuracy (Figure 1A, Figure S1A), suggesting the action of natural selection in driving the differential allele frequency distribution among human populations. Indeed, for the highest examined bin of *Q^2^* (75-87.5%) the median value for the re-sampled distribution of the enrichment statistic (see Materials and Methods) was equal to 1.065, which was found to be significantly larger than 1 (*p*<0.05).

Genic and non-genic SNPs differ in a number of different ways, most importantly in their average allele frequencies and level of linkage disequilibrium. We therefore examined directly if the observed excess could be explained by a difference in the distribution of allele frequencies, levels of population differentiation (measured as *F_ST_*) or recombination rates (and therefore linkage disequilibrium) between genic and non-genic SNPs, by directly comparing non-genic and genic SNPs with similar minor allele frequency (MAF), *F_ST_* and recombination rates. We observed an enrichment of genic SNPs for the highest bins of prediction accuracy in almost every classes of equal MAF, *F_ST_*, or recombination rate in which we divided our sample of SNPs (Table S4). This suggests that the enrichment of genic SNPs for high values of prediction accuracy is not affected by these confounding factors.

We also observed an even stronger excess of non-synonymous vs. non-genic SNPs at increasing values of prediction accuracy (Figure 1B, Figure S1B). The median value of the re-sampled distribution for the highest interval of prediction accuracy (62.5-75%) was 1.123 which is significantly greater than 1 (*p*<0.05). There was a similar enrichment of non-synonymous vs. synonymous SNPs for high values of Q^2^ (the median value of re-sampled distribution was 1.031), but this value was not significantly different from 1, possibly due to the small number of SNPs and linkage between non-synonymous and synonymous SNPs.

**Figure 2:**
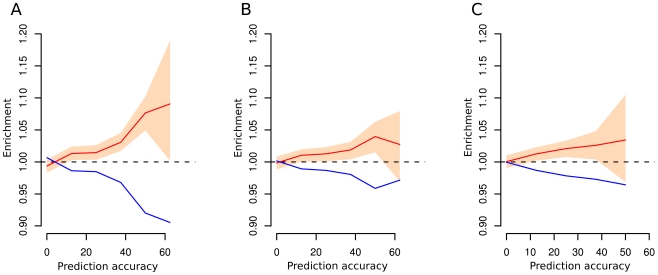
Enrichment of SNPs for different values of prediction accuracy computed on distinct models. Enrichment of genic (red line) versus intergenic SNPs (blue line) for different values of prediction accuracy computed on models comprising only pathogen diversity (panel A), subsistence strategies (panel B) and climate conditions (panel C) separately. Peach region denotes 90^th^ confidence interval computed with 1,000 bootstrap resamplings on blocks of contiguous SNPs.

We next tested whether different environmental variables differed in their contribution to allele frequency differences among populations. Using the same statistical framework, we repeated the multiple regression analysis separately using only climate, subsistence strategies or pathogen predictors for each SNP. The results show that there is a greater abundance of genic compared to inter-genic SNPs for high levels of *Q^2^* for each of the three variables (median values at last *Q^2^* bin are equal to 1.091, 1.027 and 1.034 for pathogens, subsistence or climate variables, respectively) (Figure 2). Genic enrichment at the highest bin of prediction accuracy (62.5-75%), computed by modeling the relationship between allele frequencies and environments including only pathogens predictors, is the highest among the examined factors and it is significantly greater than 1 (the lower bound of the 95% confidence interval is equal to 1.002), while the enrichment for the two other predictor classes were not significantly larger than 1.

Temperature and precipitation rate ranges have been shown to influence the biological diversity and distribution of pathogen species [47]. Nevertheless, when considering annual temperature range and annual precipitation range rather than mean levels as climate variables, we still did not observe an enrichment of genic SNPs for high values of prediction accuracy (median value at last *Q^2^* bin is 0.974).

### Quantifying the amount of selection due to adaptation to local environments

Our next goal was to identify the relative fraction of loci for which population genetic variation is significantly correlated with specific environmental factors, and to use these results to further elucidate the role played by different environmental variables in shaping human variation.

An unusually high correlation between allele frequencies and environmental variables may help identify loci involved in local human adaptation. However, these correlations are strongly affected by the non-independence of allele frequencies between closely related populations [48]. One method for circumventing this problem would be to estimate parameters of an explicit demographic model that describes the distribution of allele frequencies among populations. Unfortunately, in our case this is not computationally feasible because of the large number of populations. Instead, we assessed the relationship between the population genetic distances of each gene with at least one genotyped SNP, and a distance matrix of environmental variables via partial Mantel correlations [49], while statistically correcting for the genome-wide allele frequencies differences (Figure S2) (see Materials and Methods for further details).

**Figure 3:**
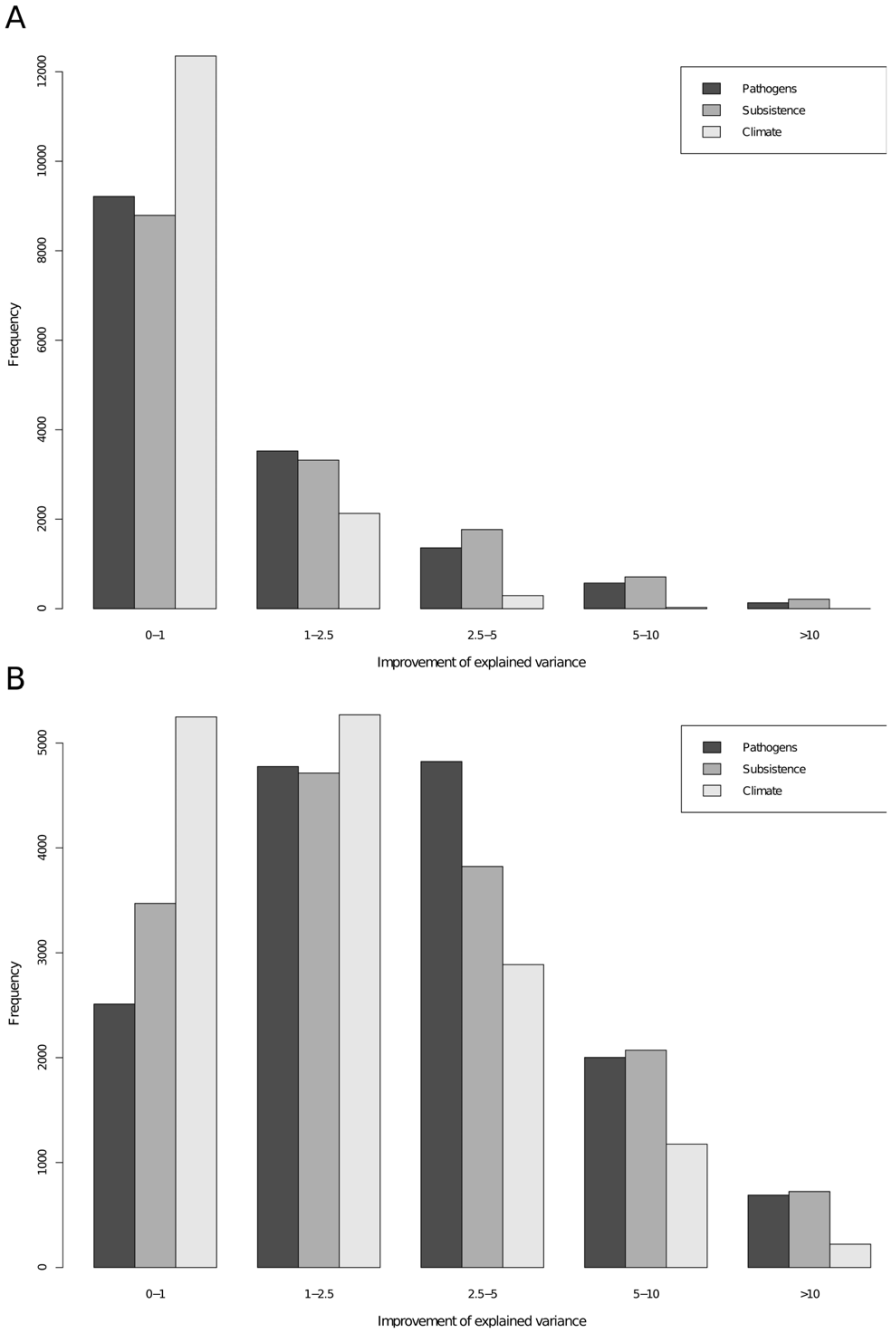
Frequencies of genes. Frequencies of genes displaying different values of improvement of explained variance with three distinct models, comprising only pathogen diversity, subsistence strategies and climate conditions, separately (A); and when testing each variable separately and taking the maximum value within each environmental category (B).

As expected, most of the genetic distance variance is explained by population demography. On average, the overall population genetic distance explains more than 95% of genetic variation for most of genes. However, the average improvement of explained variance *I*(*R* ^2^), a measure of the relative importance of each environmental variable in explaining the distribution of allele frequencies for a particular gene (see Materials and Methods), is about 1.5% for pathogen or subsistence factors, and about 0.5% for climate (Table S5). We observed a non-negligible fraction of genes (outliers in the distribution) showing highly elevated values of *I*(*R* ^2^), with some values as high as 15% (Figure 3A, Table S5). Again, such extreme values are more common for pathogens and subsistence factors than for climate, using either temperature or precipitation rate mean levels (Figure 3A) or range levels (Figure S3A).

Other environmental variables, or other quantifications of the environment, could potentially provide different results. These analyses may not have captured the main factors affecting fitness when quantifying the environment. Nevertheless, when testing each variable individually and assigning the maximum *I*(*R* ^2^) within each environmental category, we still more frequently observed high values for pathogens and subsistence factors than for climate, using either temperature or precipitation rate mean levels or range levels (Figure 3B, Figure S3B). We used a permutation procedure described in the Materials and Methods section to determine statistical significance of the *I*(*R* ^2^) values.

Examining the distribution of *p-*values, which assesses the strength of the evidence against a model in which the environmental variables do not affect allele frequencies, we observed a strikingly larger number of loci with allele frequencies significantly associated with the pathogen distance matrix (Table S5). Indeed, 103 genes show a significant *I*(*R* ^2^) value when considering the pathogen distance matrix (corrected *p-*value <0.05) while no genes were detected when considering the subsistence distance matrix or the climate distance matrix, using either temperature or precipitation rate mean levels or range levels. Again, when testing each variable individually, we observed a larger number of genes significantly correlated (corrected *p-*value <0.05) with at least one pathogen variable (229), rather than one subsistence (10) or one climate variable (9).

We also applied the Bayenv software (see Materials and Methods) to our data set and compared the results to the ones obtained using the Mantel test procedure. In general, the results are very consistent (Table S6) and the two statistics (improvement of explained variance and ranked Bayes factors) are highly correlated when testing each environmental category separately (all Spearman's rank correlation coefficients being positive, ranging from 0.1 to 0.3, with *p-*values lower than 1e-5). More importantly, among 2120 genes having at least one SNP correlated with pathogens using Bayenv, 419 are highly correlated using our method (showing an uncorrected *p-*value lower than 5e-03) and this overlap is significantly greater than expected by chance (χ^2^ test *p-*value <0.01).

When applying the Bayenv software to our data set, the number of genes having at least one SNP showing an extreme Bayes Factor value for at least one climate variable is slightly higher than the number obtained using pathogen variables (Table S6). This apparent contradictory result may be due to the fact that Bayenv tests each variable at each SNP for each locus. Therefore, for each environmental category it uses the maximum of as many different values as there are typed SNPs multiplied by the number of variables within each environmental class. Indeed, genes correlated with subsistence strategies or climate conditions (ranked Bayes Factor >0.995) show a significantly greater number of tested values for each locus than genes correlated with pathogens (one-side Wilcoxon rank sum test *p-*values of 3.62e-05 and 3.27e-06, respectively), while no difference is observed when comparing subsistence and climate variables (two-side Wilcoxon rank sum test *p-*value  = 0.62). This suggest that the discrepancy between the results using our method and the results using Bayenv is caused by the larger number of tests carried out for subsistence and climate factors in the Bayenv analysis (Table S6).

A correlation between the strength of ascertainment bias and a bias in pathogen reporting may potentially affect our results and lead to an inflation in the importance of pathogen-driven selection. To investigate this possibility, we validated our finding using low-coverage new-generation sequencing data from 1000 Genomes Project [50]. Specifically, we correlated genetic variation of more than 1,500 genes located on chromosome 1 from 9 distinct human populations with our set of environmental variables, controlling for demographic effects (see Materials and Methods). Again, we observed a fraction of genes showing highly elevated values of *I*(*R* ^2^) and such extreme values were more common for pathogens factors than for subsistence or climate predictors (Figure S4). Assessing statistical significance, we again observed a larger number of loci with allele frequencies highly correlated (uncorrected *p-*value <0.05) with the pathogen distance matrix (109), while only 0 and 11 genes were detected when considering the subsistence distance matrix or the climate distance matrix, respectively. Among the 109 genes correlated with pathogens using sequencing data, 53 exhibited a previous *p-*value, computed using genotype data, lower than 0.05 and this overlap was statistically significant (χ^2^ test *p-*value  = 1.24e-07). The latter result suggests that, despite the different number of populations and the difference in the genetic data analyzed, the results are qualitative concordant suggesting that a correlation between ascertainment bias and pathogen reporting cannot explain our results.

**Figure 4:**
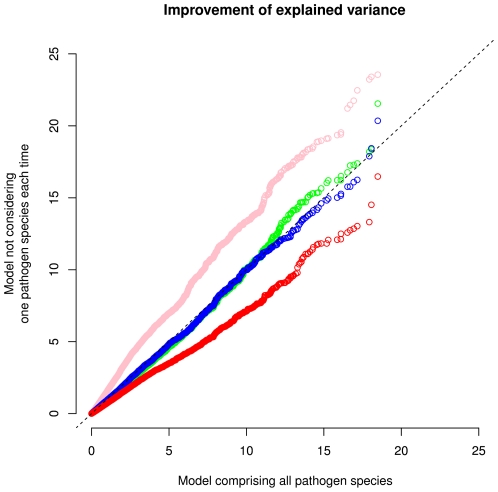
Quantile-quantile (QQ) plot of distribution of improvement of explained variance, *I*(*R* ^2^), computed with a model including all pathogen species and models not including one pathogen group. The distribution under a model not including helminthes is denoted by red, protozoa by blue, bacteria by green, and viruses by pink circles.

It is possible that different groups of pathogens have differed in their impact on human local adaptation. To test this hypothesis, we recomputed the partial Mantel correlations for each gene using different environmental matrices relating to different aspects of pathogen diversity by removing one pathogen group (viruses, bacteria, protozoa and helminthes) in the calculation of the environmental distances. By comparing the distribution of *I*(*R* ^2^) for the model with all pathogen species (*I*(*R* ^2^)_FULL_) to the ones missing one of the pathogen groups (e.g. virus diversity, *I(R^2^)*_w/o VIRUS_) it is possible to evaluate the impact of the missing pathogen group on the relationship between genetic variation and pathogen diversity. The reduction in *I*(*R* ^2^) when not considering a particular pathogen group provides a measure of the relative impact of this particular group in explaining local adaptation. QQ plots showing the difference between including all pathogens and dropping one of them are illustrated in Figure 4. Clearly, removing helminth diversity from the model leads to a drastic decrease in the distribution of *I*(*R* ^2^) (Figure 4). Conversely removing virus diversity from the pathogen distance matrix results in an apparent increase in *I*(*R* ^2^), presumably because the matrix then is more strongly dominated by the helminth distances.

### Biological significance of signals of human adaptation to pathogens

The above results demonstrate that pathogens diversity is a major factor in human adaptation to local environments. We further examined the genes whose genetic variation is correlated with the pathogen diversity matrix using analysis of Gene Ontology (GO), by testing whether certain ontology terms are enriched for SNPs correlated with pathogen diversity.

By investigating statistically enriched terms in our set of pathogen-associated SNPs, we found an over representation of shared GO terms relating to regulation of immune system, defense and inflammatory response (Table 1, Table S7). In addition to these more general ontology categories, we found enrichment in categories related to mechanisms involving a direct response to external agents or host-pathogen interaction (e.g. response to wounding, JAK-STAT cascade, antigen binding, oxidoreductase activity, endosome).

**Table 1:** ontology analysis results (Process domain) for SNPs mapping on genes that correlate with pathogen diversity.

Ontology term	Number of hits	*p-*value
Process		
defense response	29 Hits At 22 Loci	3.10e-04
response to biotic stimulus	29 Hits At 22 Loci	2.30e-03
immune response	27 Hits At 21 Loci	4.90e-04
response to wounding	14 Hits At 11 Loci	2.73e-04
DNA replication and chromosome cycle	13 Hits At 10 Loci	4.60e-03
negative regulation of cell proliferation	11 Hits At 7 Loci	4.70e-03
inflammatory response	9 Hits At 7 Loci	4.33e-03
innate immune response	9 Hits At 7 Loci	4.92e-03
viral life cycle	6 Hits At 4 Loci	1.64e-04
pyrimidine nucleotide metabolism	6 Hits At 4 Loci	2.79e-04
cytosolic calcium ion concentration elevation	5 Hits At 3 Loci	8.94e-05
pyrimidine nucleotide biosynthesis	4 Hits At 3 Loci	6.94e-03
JAK-STAT cascade	4 Hits At 3 Loci	5.50e-03
viral genome replication	4 Hits At 3 Loci	1.77e-03
viral infectious cycle	4 Hits At 3 Loci	6.26e-03

We further interrogated the KEGG PATHWAY database, focusing our attention on hierarchical categories related to the immune system or immune diseases. We identified 3 KEGG pathways showing a significantly higher than expected number of pathogen-associated genes as determined by a bootstrap procedure (Table 2). Two of the identified KEGG pathways are related to immune-related signaling processes (Leishmaniasis pathway and Toll-like receptor signaling pathway), while the remaining one involves allograft rejection.

**Table 2:** pathways enriched with genes which correlated with pathogen diversity.

KEGG Pathway	Enrichment p value	Number of pathogen-associated genes	Pathogen-associated genes
Allograft rejection	0.0011	5	*CD28, FAS, IFNG, IL10, IL4*
Leishmaniasis	0.0075	13	*C3, FCGR2A, FOS, IFNG, IFNGR2, IL10, IL1B, IL4, JAK1, MAPK13, MYD88, NFKBIB, RELA*
Toll-like receptor signaling pathway	0.046	9	*CCL4, FOS, IL1B, IL6, MAP3K7IP1, MYD88, RAC1, RELA, SPP1*

It has previously been argued that SNPs associated with susceptibility to complex diseases, or other important phenotypic traits, are more likely to be targets of natural selection than random genes [27], [38]. To test this hypothesis, we extracted a collection of more than 2,000 SNPs which have been associated with specific phenotypic traits and/or diseases and typed in on our panel from the GWAS database (www.genome.gov).

**Figure 5:**
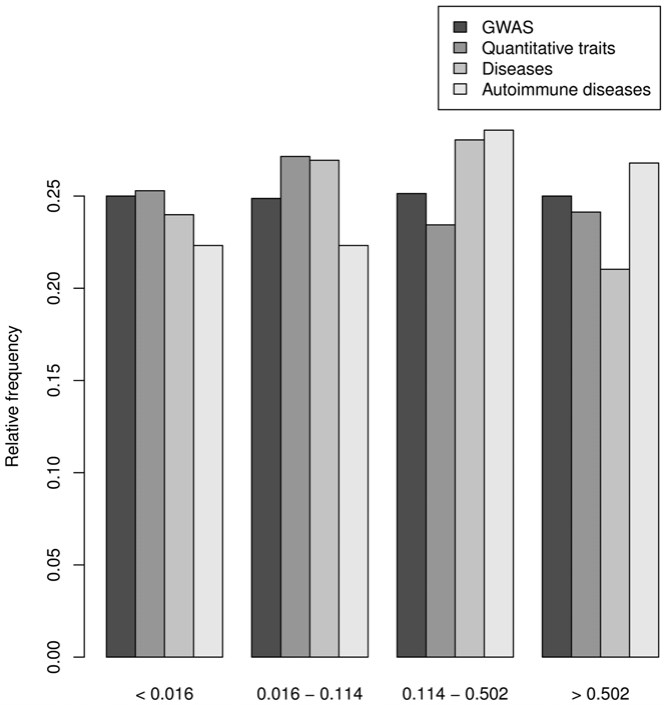
Frequencies of genes for different values of improvement of explained variance. Classes were defined as quartiles of distribution of improvement of explained variance for genes associated to a trait or a disease in GWASs. Bars are colored according to the association to a quantitative trait, a disease or an autoimmune disease.

To elucidate the biological impact of the inferred local selection, we compared -log_10_ of the *I*(*R* ^2^) *p*-values in a set of 770 genes containing at least one significant SNP from a GWAS study. Genes were divided into three categories depending on whether the SNPs were associated with an autoimmune disorder, another disease, or a quantitative trait. We use the *p*-values as more appropriate measures of the strength of the evidence rather than the *I*(*R* ^2^) themselves. Genes associated with autoimmune diseases show a clear increase in the proportion of genes with low *I*(*R* ^2^) *p*-values (large -log_10_ values; Figure 5).

We then investigated which autoimmune diseases more often have been targeted by natural selection. Similarly to our previous Gene Ontology analysis, we identified celiac disease (susceptibility), ulcerative colitis (susceptibility), multiple sclerosis (susceptibility, severity or age of onset), and type 1 diabetes (susceptibility) (Table 3), as the most common disease categories.

**Table 3:** correlated with pathogen diversity which have been previously associated to susceptibility to autoimmune diseases.

Disease	Associated genes
Celiac disease	*TNFRSF9, CCR4, ETS1, CD28, SH2B3, REL, CIITA, LPP, SLC9A4*
Ulcerative colitis	*IL10, IFNG, CIITA, FCGR2A, IL19, REL*
Type 1 diabetes	*DLK1, SH2B3, CTSH, IL10, C16orf75*
Multiple sclerosis (susceptibility, severity or age of onset)	*CBLB, RPL5, KCNB2, CENPC1, FUT8*
Systemic lupus erythematosus	*ETS1, SOCS6*
Rheumatoid arthritis	*REL, SH2B3*
Vitiligo	*LPP, RERE*
Crohn's disease (or combined with sarcoidosis)	*FUT2*
Behcet's disease	*IL10*
Ankylosing spondylitis	*ANTXR2*

## Discussion

In recent years, great efforts have been made to assess the role played by natural selection during human evolution [2], [3], [17], [19], [46], [48]. Genome-wide scans for recent positive natural selection identified a putative list of non-neutrally evolving genes involved in specific biological pathways including metabolism, immune function, and skin pigmentation [4], [7]-[16]. These findings suggest that selective pressures related to adaptation to local environmental conditions might have contributed in shaping human genetic variation.

Here we developed a statistical framework for identifying signals of adaptation to local environments. We correlated the spatial allele frequency distribution of a large sample of SNPs, genotyped in more than 50 populations distributed worldwide, to a set of environmental factors, describing local geographical features such as climate conditions, diet regimes (measured as subsistence strategies) and pathogen loads.

Previous studies have shown that SNPs with an increased degree of population genetic differentiation (measured using *F_ST_* or other statistics) are enriched for genic SNPs [14], [46]. Our analyses confirm these observations by finding a significant enrichment of genic SNPs, in particular non-synonymous SNPs, vs. intergenic SNPs for high values of the regression prediction accuracy, *Q^2^* (Figure 1). *Q^2^* provides a measure of genetic differentiation among populations relative to the defined set of environmental variables.

Interestingly, the enrichment of non-synonymous SNPs is quantitatively greater than the enrichment of genic variants, in line with the hypothesis that a larger fraction of non-synonymous SNPs has direct functional effects.

We can exclude the possibility that this enrichment is explained by different distributions of recombination rates, allele frequencies or *F_ST_* between genic and intergenic SNPs, as the enrichment is also apparent when stratifying according to MAF, recombination rate or *F_ST_*.

It is worth noting that the SNP data analyzed here suffer from an ascertainment bias, owing to the protocols used for selecting SNPs for the genotyping platforms. One of the main effects of the ascertainment bias is a shift toward common variants with non-negligible consequences on various statistics, such as measures of population structure [51]-[54]. However, as previously mentioned, the results hold up even when stratifying with respect to MAF and *F_ST_*, suggesting that ascertainment biases, which primarily affect the data through the allele frequency, do not have a strong effect on our results.

Overall these results strongly indicate that the enrichment of genic and nonsynonymous variants among SNPs with a high value of *Q^2^* may truly reflect the action of natural selection.

Importantly, we find a quantitatively higher, and statistically significant, enrichment of putative functional SNPs for high values of *Q^2^* for models comprising pathogens as predictors rather than climate or diet (Figure 2), even we testing for additional climate variables such as temperature and precipitation annual ranges. Although all the environmental factors we have investigated contribute to *Q^2^*, our results suggest that pathogens are a more important driver of local adaptation than other factors explored in this paper.

To further investigate this issue, we computed partial Mantel correlation between the locus-specific population genetic distance and three different matrices describing pathogen load, diet regimes or climate conditions. In doing so, we used the average distance of allele frequencies as a covariate to control for background demographic processes. As expected, most (approx. 95%) of the variance in allele frequencies among populations can be explained by non-adaptive processes. Nonetheless, we were able to identify a non-negligible contribution of selection. Several loci showed large values (>15%) in the improvement of explained variance *I(R^2^)*, when adding a specific environmental matrix (pathogen, diet or climate; see Materials and Methods; Figure 3, Figure S3, Table S5).

Genes with a statistically significant *I(R^2^)* are likely targets of local selection because *I(R^2^)* measures the increase in explained variance by an environmental factor when taking average distances among populations into account. In particular, there is a strikingly larger number of genes significantly correlated to the distance matrix describing pathogen diversity compared to the ones related to climate conditions or diet regimes. A total of 103 genes are significantly correlated in frequency with pathogen predictors while none correlates with climate or subsistence strategies. This predominant role of pathogen-driven selection in the human genome is confirmed when testing each variable within each environmental category separately (229, 10 and 9 genes significantly correlated in frequency with at least one pathogen, subsistence and climate variable, respectively).

Furthermore, we validated our results using low-coverage sequencing data for a smaller set of SNPs and populations, ruling out the possibility that ascertainment bias coupled with a bias in reporting pathogen diversity may lead to the observed prevalence of pathogen-driven selection. We should add that other factors could affect local adaptation than the factors examined here. The quantitative measures used here may not be the ones that correlate most closely with the components of the environment that affect fitness. Other measures of local climate or subsistence, that include variables not examined here might show a stronger effect on local adaptation. However, among the quantitative measures of environmental factors explored here, it is clear that pathogen load has been the most important factor shaping human genetic diversity.

It is perhaps not surprising that selection related to pathogens appears to be the most dominating driver of local adaptation, given the number of studies reporting pathogen related selection in humans, including selection on proteins used by pathogens to infect cells (such as certain blood group antigens [25], [55]), pathogen receptors (such as the TLR family [30], [31] and glycosylated extracellular membrane proteins [56]) and selection on genes product directly involved in immune/defense response to pathogens (e.g. [26]-[28], [33], [57]-[62]).

Infectious diseases have represented, and still represent, one of the major causes of death for human populations, especially in developing countries [63], [64]. Not surprisingly, genes responsible for heritable variation in the response to pathogens are likely targets of natural selection.

It may be more surprising that the pressure imposed by parasitic worms (helminthes) on human genes has been stronger than the one due to viral, protozoa or bacterial agents (Figure 4). Perhaps this is due to the fact that helminthes evolve slower than unicellular/viral agents and that they often have complex life cycles which results in a relatively stable geographic distribution [65]. Evolutionary changes in the helminthes, therefore, occur at a similar time-scale to that of humans, allowing for a true co-evolutionary interaction between humans and the pathogen. Faster evolving species (e.g., viruses) may perhaps not exert the same selective pressure for long enough time to induce a sufficiently strong change in allele frequencies.

We identified signatures of pathogen-mediated selection in 103 distinct human genes. Overall, genes highly correlated with pathogen diversity show a significant enrichment of immunity related functions, according to Gene Ontology analysis (Table 1). Again these findings strongly suggest that the candidate loci we detected truly are targeted by natural selection due to adaptation to pathogens.

Among 103 genes targeted by pathogen-driven selection, 23 are directly related to immunity processes, according to ImmPort database (www.immport.org). These genes encode signaling molecules involved in the inflammatory response, such as *IL6*, *LRRC19,* and *PON2*, cell surface proteins participating in immune functions, such as *ADAM17, ITGAL,* and *LAG3*, and signal transducers of the innate and adaptive immune response such as *MYD88* (Table S8). In particular, *ADAM17* has been shown to be involved in viral entry and to participate in intestinal inflammation triggered by Toll-like receptors (TLRs). In addition to *ADAM17*, we have identified 9 other genes with high *I*(*R* ^2^) values when using pathogen diversity as covariate that also participate in the Toll-like receptor signaling pathway (Table 2). One of these genes, *MYD88,* encodes a cytosolic adapter protein central for the transduction of the immune response. This protein is implicated in sensing retroviral infections by endosomes [66]. *MYD88* is also implicated in the immune response to *Bacteroides fragili*s [67], *Plasmodium berghei* [68] and helminth infections [69]. Several of the 23 immunity-related genes with high *I(R^2^)* values have previously been reported to be related with pathogen infection, mainly to bacterial infections and viral infections (Table S8).

Interestingly, the two enriched signaling pathways we identified relate to two very different categories of immune response and they function in the defense against different pathogen groups (Table 2). Toll-like receptors (TLR) are molecules involved in the innate immunity and account for the first-line defense against viruses, bacteria, fungi and protozoa (reviewed in [70]), although previous studies have demonstrated the TLR-mediated signaling pathway is also important for resistance to helminthes in mice (Schistosomal-derived lysophosphatidylcholine is involved in eosinophil activation and recruitment through Toll-like receptor-2-dependent mechanisms).

While different TLRs have previously been shown to be targets of natural selection [30], our data indicate that pathogens have also exerted a pressure on genes that impinge on the cellular pathways associated with these receptors.

The second signaling pathway enriched with 13 genes targeted by pathogen-driven selection genes is Leishmaniasis (Table 2). Leishmania are obligate intracellular parasites (protozoa) that produce diseases in humans and mice. When associated with malnutrition, Leishmania infection can produce extremely serious symptoms, and a recent WHO survey indicates that epidemics of visceral leishmaniasis can lead to massive deaths in affected areas (http://www.who.int/leishmaniasis/). Thus, the parasite is likely to have exerted a strong selective pressure during human evolutionary history.

Dendritic cells (DC), sentinels of the immune system, detect Leishmania in vivo. It has been shown that MyD88-dependent receptors are implicated in the direct recognition of Leishmania by DC [71], [72], pointing again to MyD88 as an important element in host-pathogen recognition.

Genes related to immunity and inflammation regulation are known to be common targets of natural selection [38]. In particular, recent reports have suggested that a portion of susceptibility alleles for autoimmune diseases might be maintained in human population because they confer increased resistance against infection [27], [38], [43]. The identification of several autoimmune disease-related genes as target of natural selection may be consistent with the hygiene hypothesis [73]. This model states that humans have adapted to a pathogen-rich environment that no longer exists in industrialized societies. This change has reduced the exposure of the immune system to antigens, causing an overreacting immune response which favors the development of chronic inflammatory conditions [73].

Indeed, our data indicate that SNPs with allele frequencies that correlate highly with pathogen variables are enriched for GWAS SNPs associated with autoimmune diseases (Figure 5). Specifically, among our candidate genes we identified several loci that have been associated with celiac disease, ulcerative colitis (UC), type 1 diabetes (T1D), Crohn's disease (CD), and multiples sclerosis (MS) (both susceptibility and disease severity) (Table 3). Signatures of natural selection at risk alleles for celiac disease, UC and CD have previously been described [27], [38], although these variants were located in genes different from the ones we describe herein. Conversely, only a minority of genes involved in the susceptibility to T1D and MS have been described as possible selection targets [38], although a certain degree of overlap among genes involved in MS pathogenesis and loci subjected to virus-driven selection has previously been noticed [40]. Therefore, our data further support the notion that natural selection has contributed to shaping the pattern of genetic variability relating to this common disorder.

Hancock and colleagues recently performed a genome-wide scan for selection signals by detecting SNPs strongly correlated in frequency with climate [74]. They investigated genetic variation in a similar set of populations, and a similar data set of genotyped SNPs as this study. They retrieved a number of SNPs putatively subjected to climate-mediated selection, while we found only weak signals for genetic adaptation to climate conditions. There are several possible reasons for this apparent discrepancy. First, Hancock and colleagues' and our method are intrinsically different both in the analyzed elements (SNPs rather than genes, respectively) and in the approach to detecting significant signals (extreme Bayes Factors versus *p-*values, respectively). Most likely, our criterion for selecting extreme genes is more conservative than the one used by Hancock and colleagues. However, when applying their approach to our data set, we retrieved a significant overlap of genes correlated with different environmental factors (Table S5, Table S6). These observations suggest that the two studies, although examining different climate variables in a different sample of populations, lead to concordant results. Second, they found evidence of selection for SNPs located in immune-related genes or previously associated with autoimmune diseases and inflammatory conditions. As stated by authors themselves, it is likely that the selective pressure imposed on these genes is related to pathogen resistance/susceptibility [74], which is in agreement with our main results.

A major assumption in this study, is that the number of different pathogen species (pathogen richness or diversity) transmitted in a given geographic location is a good estimate of the pathogen-driven selective pressure for populations living in that area [25], [39]. Indeed, there is evidence that pathogen richness is a suitable and more effective measure than standard epidemiological parameters (like prevalence or mortality) for estimating the selective pressure exerted by infection agents, and that it better captures the signatures left by adaptation to specific pathogens throughout recent human evolution [27], [40], [41]. It is worth noting that our measure of pathogen evolutionary is noisy, discrete, possibly affected by report biases and calculated on a country level.

More accurate worldwide epidemiological data, as well as more detailed description of diet regimes for human population, are required to obtain a clearer picture of the effect of genetic adaptation to pathogen load or subsistence strategies, especially when comparing with adaptation to climate.

However, any inadequacies of the statistics we use to measure pathogenic environment will lead us to underestimate the role of the pathogenic environment in human local adaptation. Perhaps pathogen related selection plays an even stronger role in human evolution than what has been evidenced in this study and in previous studies.

## Materials and Methods

### Genetic variation data

We investigated the spatial distribution of allele frequencies using genotype data for 55 distinct human populations, comprising more than 1,500 individuals, by joining data from the Human Genome Diversity Panel (HGDP-CEPH) [75], [76] and from HapMap Phase III [77], not considering admixed populations (Table S1).

A total of more than 500k SNPs were analyzed after removing those not covered in both panels and those located on sex chromosomes (Table S2). We used the folded frequency spectrum of genotyped SNPs to quantify allele frequencies.

Two categories of SNPs were considered: genic and intergenic. SNPs were defined as genic if they were located in transcribed regions or were no further than 500 bp upstream transcription start sites. SNPs were defined as intergenic if they were located in a region larger than 100 kbp containing no annotated gene, according to USCS Genome Browser database of gene predictions based on data from RefSeq, Genbank, CCDS and UniProt (http://genome.ucsc.edu). In case of multiple isoforms, the longest transcript was used. A total of 225,502 and 216,151 polymorphisms were classified as genic and intergenic, respectively. A total of 14,804 genes containing at least one genotyped SNP was retrieved.

Data from the 1000 Genomes Project [50] were retrieved from the dedicated website (http://www.1000genomes.org/). Low-coverage SNP genotypes for each one of the nearly 1,5 K analyzed genes located on chromosome 1 were organized in a MySQL database. Populations from countries not included in the HGDP-CEPH panel were excluded. A total of 727 unrelated individuals belonging to 9 distinct populations located in 8 different countries were analyzed. A set of programs was developed to retrieve genotypes from the database and to analyze them according to selected populations. These programs were developed in C++ using the GeCo++ [78] and the LibSequence [79] libraries.

### Environmental variables

We defined a set of environmental variables for each country from which SNP data were available. Previous studies examining signatures of adaptation to local climates, selected specific variables to represent the physiological effects of different climates on humans [21]. Similarly, adaptation to diet has been investigated by examining correlations between genetic variation and different subsistence strategies among populations [22]. Finally, previous studies have suggested that pathogen diversity (i.e. the number of the different pathogen species transmitted in a given geographic location) is a reasonable proxy for the selective effects exerted by pathogens in an area [25], [39]. Based on these previous studies, we chose a total of 14 environmental variables as proxies for local environmental conditions (Table S3), including climatic and geographic factors (distance from the sea, mean annual temperature, mean annual precipitation rate, mean annual relative humidity, mean annual short wave radiation flux), subsistence strategies (relative amount of human activity spent in agriculture, animal husbandry, fishing, hunting, gathering) and pathogen diversity (number of different species of viruses, bacteria, protozoa, helminthes). Additionally, we used annual temperature range and annual precipitation rate rather than annual mean levels as supplemental climatic factors.

We obtained climatic data from the NCEP/NCAR Reanalysis Project database (http://www.esrl.noaa.gov/psd/data/reanalysis/reanalysis.shtml) by averaging annual values across the last 50 years. Subsistence strategy data were collected from Murdock's Ethnographic Atlas (1967): for each population we retrieved the percentage of activity spent in each of the examined subsistence activities. For three populations we could not assess an unambiguous set of subsistence values. The number of different pathogen species (pathogen richness) was retrieved from the Gideon database (http://www.gideononline.com/), as in references [25], [27], [40]-[42]. Cases of transmission due to tourism and immigration were not included; thus, only species that are transmitted within the countries were included. However, species that recently have been eradicated, for example, as a result of vaccination campaigns, were recorded as present in the country.

### Multiple regression analyses

We applied a Projection to Latent Structures (*PLS*, also known as Partial Least Squares) multiple regression [80] to model the relationship between population allele frequencies of each SNP and a matrix describing environmental factors. This algorithm can handle highly correlated predictors and can effectively separate the weight of each predictor in the multiple correlation even in case of strong collinearity among variables (which is likely to be the case for environmental factors) [80], [81]. For the model including all the environmental factors we applied an Uninformative Variable Elimination algorithm [82] before the regression. In this way we could greatly increase prediction accuracy by not considering predictors with very low regression coefficients.

For each SNP we assessed the relationship between population allele frequencies matrix (*F*) of dimensions *55x1* and environmental predictors matrix (*M*) of *55x14* dimensions. *F* describes minor allele frequency at each population for the examined SNP, whereas *M* describes all the 14 environmental variables for each population. For each regression we computed the cross-validated prediction accuracy (*Q^2^*), estimated by a leave-one-out procedure, as:
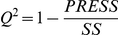
where *PRESS* is the Predictive Residual Sum of Squares calculated from the models obtained on the reduced data of the leave-one-out procedure, and *SS* is the Sum of Squares of *F* corrected for the mean. Formally, *PRESS* is the sum of squares of the differences between observed and predicted responses:
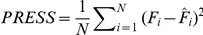
and thus is a measure of the predictive ability of the model, whereas *SS* is computed as:
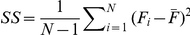
where *N* is the number of observations (55 populations in our case), 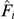 is the predicted response at the *i^th^* population and 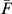 is the mean value for the response matrix (see [80], [83], [84] for further mathematical aspects of *PLS* regressions and model parameters estimation).

*Q^2^* provides a measure of how well a model predicts the observed data using a cross-validation procedure, based on a partitioning of the sample into complementary subsets of observations. Iteratively each of the partitions are treated as a training set and the level of fit of the model is computed by using the remaining partition as a validation set. In our case, *Q^2^* measures how well a model with environmental variables as covariates predict the observed distribution of allele frequencies among populations. We use it as a measure of population subdivision along the axes defined by the environmental variables. If populations which differ strongly in terms of environmental variables also differ strongly in terms of allele frequencies, *Q^2^* will be large. If allele frequencies do not covary with the environmental variables, *Q^2^* will be small.

### Enrichment of genic SNPs

We divided the distribution of *Q* ^2^ values into bins, and for each bin we calculated the relative enrichment of genic, non-synonymous and intergenic SNPs, as previously proposed [46]. Enrichment is here defined as the proportion of genic/non-synonymous (or intergenic) SNPs in the bin divided by the total proportion of genic/non-synonymous (or intergenic) SNPs. We applied a Moving Block Bootstrap (MBB) re-sampling procedure to correct for the non independence among loci taking into account the possibility of an increased level of linkage disequilibrium (LD) near the positively selected site [85], [86]. The MBB method consists of drawing blocks of fixed length uniformly at random and with replacement and joining them to form a new sequence. Standard deviations are estimated from the distribution of bootstrap values. Critical values used in hypotheses testing were obtained directly from the quantiles of the distribution. We performed this procedure 1,000 times for each chromosome separately by creating *(n+b-1)* overlapping blocks (where *n* equals to number of polymorphic sites and *b* equals to block size) and drawing *n/b* blocks. Block sizes were set to 40 and 100 contiguous SNPs corresponding to segments of approx. 200 k bp and 500 k bp on average, respectively. Bins with less than 100 SNPs were merged with the immediately lower bin. The results were qualitatively similar using 40 and 100 SNPs, and only the results for 40 SNPs are discussed in the main text. The results for 100 SNPs are also provided in Figures S1, S2, S3, S4.

*F_ST_*, a measure of population genetic difference, was estimated as previously proposed [87]. Recombination rates were obtained from the UCSC Genome Browser (table ‘recombRate’) as estimates computed in 1 M bp intervals based on the deCODE maps [88].

### Partial correlations

Nearly 15,000 genes containing at least one genotyped SNP were retrieved, according to UCSC Genome Browser database (http://genome.ucsc.edu). For each retrieved gene we computed population genetic distances, as proposed by Reynolds and colleagues [89]. Distances between the environmental values in different locations were calculated as Euclidean distances, which correspond to the square distances between the two vectors of environmental variables. The latter have been scaled to unit variance to ensure that the dissimilarity matrices are not to be dominated by the variables with the largest variance. The dissimilarity matrices were then obtained by averaging distances over each variable within each environmental category (pathogen diversity, subsistence strategies and climate conditions).

For each gene we assessed the relationship between the locus-specific population genetic distances matrix (*Y*) and the distance matrix for environmental variables (*X*) via partial Mantel correlations [49]. Each row or column in *X* and *Y* correspond to a population. *Y* contains locus specific population genetic distance values and X contains environmental distances. Partial Mantel correlations are a non-parametric statistical procedure for quantifying association between two distance matrices, while controlling for the effect of a third distance matrix. The latter independent distance matrix, *Z*, is here the overall population genetic distance among populations [89] computed over all loci. As shown in Figure S2, the Z matrix reflects the general patterns of human population structure.

Our procedure, therefore, accounts for the non-independence of populations and controls, at least in part, for the correlations caused by standard neutral demographic processes (Figure S2). For each variable we calculated the improvement of explained variance [90], here called *I*(*R* ^2^), and used this as a measure of the relative importance of each environmental variable in explained the distribution of allele frequencies for a particular gene.

*I*(*R* ^2^) is calculated as:
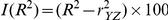
where *R* ^2^ is the explained variance of the model, estimated as:



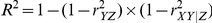

*r_YZ_* is the correlation coefficient between *Y* and *Z*, while *r_XY/Z_* is the partial correlation coefficient between *X* and *Y* given *Z* defined as:



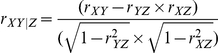
Again *r_XY_* is the correlation coefficient between *X* and *Y* while *r_XZ_* is the correlation coefficient between *X* and *Z.*

Statistical significance was assessed by permuting rows (populations) for the dependent matrix (*Y*) and recalculating our statistic for the permuted data. Rows were permuted only within the same stratus (continent). *P-*values are then calculated as the fraction of permuted values that are greater than the observed value of the statistic. This imposes the need for a large number of permutations when estimating small *p-*values which is computationally expensive and unfeasible for some problems. To reduce computational time we computed approximate *p-*values using a previously proposed asymptotic method [91]. Briefly, *p*-values were computed by approximating the right tail of the distribution of permuted statistics by a Generalized Pareto distribution. Parameters of the Pareto distribution are fitted using either the asymptotic maximum likelihood method or a ‘combined method’ previously proposed [92]. *P*-values were corrected for multiple testing using a procedure which controls the false discovery rate under dependence assumptions [93]. For partial correlations, we considered only 52 populations to which we could unambiguously assess all environmental variables (Table S3). We performed the same procedure when using low-coverage new-generation sequencing data from the 1000 Genomes project, with the Z matrix, the overall population genetic distance among populations (see above), computed by averaging distance matrices over all loci.

We also used the method by Coop and colleagues [94] to calculate Bayes factors relating to the effect of an environmental variable on the geographic distribution of allele frequencies. For each SNP a Bayes factor is calculated, providing a measure of the increase in fit of a model with a linear relationship between allele frequencies and an ecological variable over a null model. A Gaussian model is assumed with a covariance matrix of allele frequencies among populations estimated from a sub-sample of segregating sites. SNPs which are outliers in the empirical distribution of Bayes factors may possibly be affected by local selection in response to the environmental variable. The covariance matrix was estimated on 1,000 random polymorphisms. SNPs were ranked according to their Bayes factor after dividing them in 10 classes of similar minor allele frequency. A significance threshold for ranked Bayes factors was set to 0.995.

Both algorithms use the empirical distribution of genome-wide genetic distances between populations as a null model. A major difference between the methods, in addition to the fact that one is Bayesian and the other frequentist, is that the partial correlation approach used here combines the information from all SNPs in a region, or in a gene, whereas the approach by Coop *et al.* [94] performs the analyses SNP by SNP. In addition, our method is non-parametric and does not rely on assumptions of normality. The degree to which either of these approaches are preferable depends perhaps on the degree to which the parametric assumptions are met by the data. Our objective here is not to compare the two methods, which were developed in parallel. However, we have included results based on the methods of Coop *et al.* [94] to show that our conclusions are not dependent on the specific choice of statistical method.

All computation were performed in the R environment using the following packages: vegan, pls, gPdtest, VGAM. All data and scripts used are available at http://bioinformatics.emedea.it.

### Gene ontology and GWAS analyses

Gene ontology (GO) analyses were performed with GONOME [95], an algorithm that identifies GO terms over-represented in a set of genomic position (in our case SNPs) compared to that expected in random positions. GONOME avoids biases toward GO terms linked to larger genes or genes having more genotyped SNPs and also can take into account non-independence between linked sites by setting an appropriate cluster distance value (50 k bp in our case).

Analyses of functional pathways were investigated querying the KEGG database (www.genome.jp/kegg) in the following hierarchical categories: Immune system, Immune system diseases, Neurodegenerative diseases, Infectious diseases. Enriched pathways were retrieved as having a significantly higher than expected number of associated genes using 10,000 bootstrap samples. For GO analyses we considered pathogen-associated genes to be those having an uncorrected *I*(*R* ^2^) *p-*value lower than 5e-3 in order to increase the number of analyzed loci and thus statistical power.

Genome-wide association studies (GWAS) data were retrieved from A Catalog of Published Genome-Wide Association Studies [96] (www.genome.gov), updated on December 1^st^, 2010.

## Supporting Information

Figure S1 Enrichment of genic (red line, panel A) or non-synonymous (red line, panel B) vs. intergenic SNPs (blue line, both panel) for different values of prediction accuracy. Peach region denotes 90^th^ confidence interval computed with 1,000 bootstrap resamplings on overlapping blocks of 100 contiguous SNPs.
(EPS)

Figure S2 UPGMA (Unweighted Pair Group Method with Arithmetic Mean) clustering of populations according to the overall population genetic distance matrix. Each node of the dendogram represents a distinct population and is labeled regarding its continental origin (AF: Africa, OC: Oceania, EA: East Asia, CA: Central Asia, EU: Europe, AM: America).
(JPG)

Figure S3 Frequencies of genes displaying different values of improvement of explained variance with three distinct models, comprising only pathogen diversity, subsistence strategies and climate conditions (including annual temperature and precipitation rate ranges instead of mean levels), separately (A); and when testing each variable separately and taking the maximum value for each environmental category (B).
(EPS)

Figure S4 Frequencies of genes on chromosome 1 displaying different values of improvement of explained variance with three distinct models, comprising only pathogen diversity, subsistence strategies and climate conditions, using low-coverage new-generation sequencing data from 1000 Genomes Project.
(EPS)

Table S1 List of sampled populations with specification of their country, continental group and number of genotyped individuals.
(XLS)

Table S2 Chromosomal location for the entire set of analyzed SNPs.
(XLS)

Table S3 Environmental variables for each population. Values were averaged for each country.
(XLS)

Table S4 Enrichment of genic SNPs for different classes of similar Minor Allele Frequencies (MAF), *F_ST_* or recombination rate.
(PDF)

Table S5 Improvement of explained variance (termed *I(R^2^)*) values and their *p-*values for the whole set of genes.
(XLS)

Table S6 Genes having at least one significantly associated SNP with Bayenv software (ranked Bayes factor >0.995) for one variable for each environmental class.
(XLS)

Table S7 Gene ontology analysis results (Function and Component domains) for SNPs mapping on genes which correlate with pathogen diversity.
(PDF)

Table S8 Annotation of genes correlated with pathogen diversity.
(XLS)
