# Correction: Signatures of Environmental Genetic Adaptation Pinpoint Pathogens as the Main Selective Pressure through Human Evolution

**DOI:** 10.1371/annotation/ca428083-dbcb-476a-956c-d7bb6e317cf7

**Published:** 2011-11-21

**Authors:** Matteo Fumagalli, Manuela Sironi, Uberto Pozzoli, Anna Ferrer-Admetlla, Linda Pattini, Rasmus Nielsen

The fourth author's name was misspelled. The correct name is Anna Ferrer-Admetlla. The correct citation is: Fumagalli M, Sironi M, Pozzoli U, Ferrer-Admetlla A, Pattini L, et al. (2011) Signatures of Environmental Genetic Adaptation Pinpoint Pathogens as the Main Selective Pressure through Human Evolution. PLoS Genet 7(11): e1002355. doi:10.1371/journal.pgen.1002355 

