# Supplementary figures and images for: Signatures of Environmental Genetic Adaptation Pinpoint Pathogens as the Main Selective Pressure through Human Evolution
Source: PLoS Genet. 2011 Nov 3;7(11):e1002355. doi: 10.1371/journal.pgen.1002355 (PMC3207877; doi:10.1371/journal.pgen.1002355)

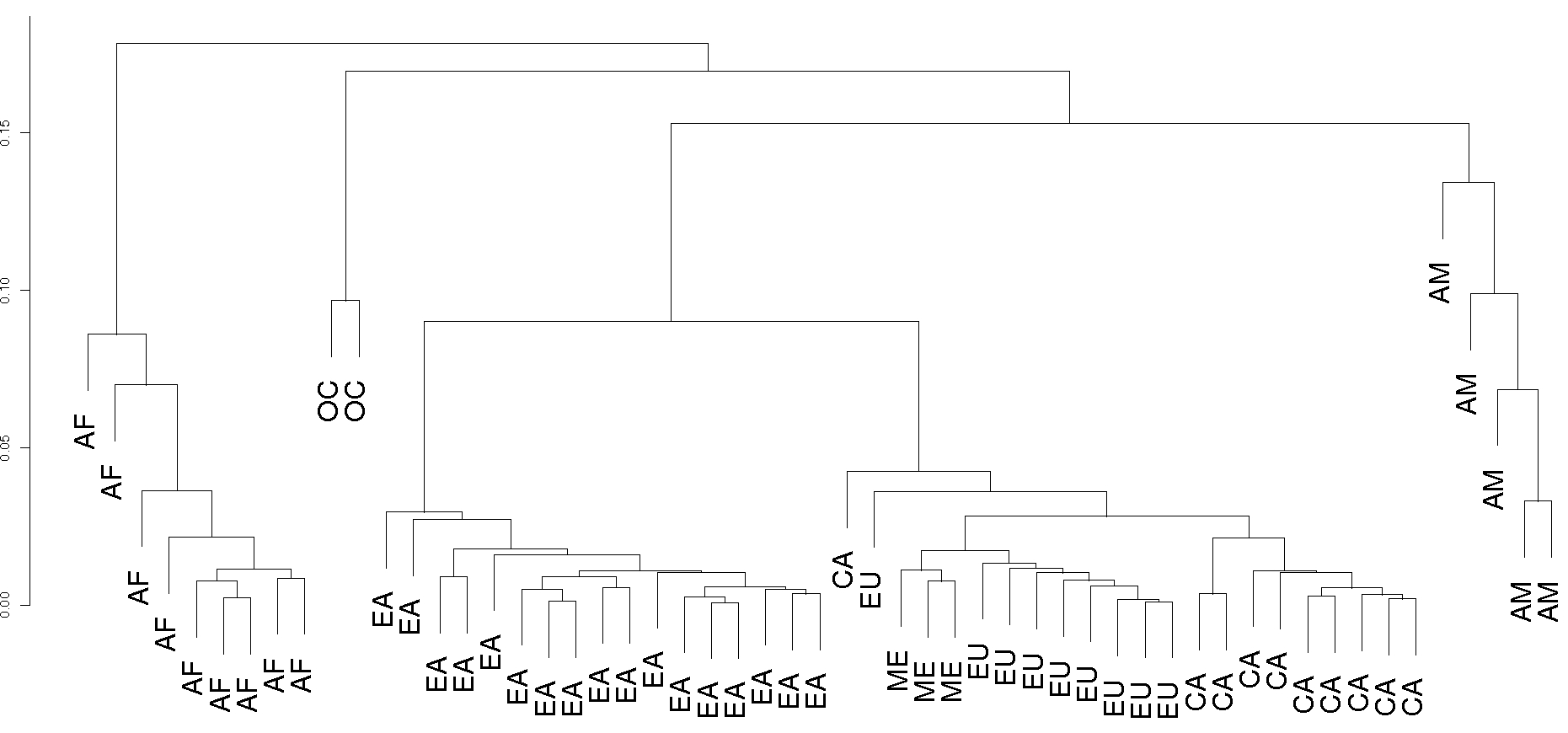

Supplement: Figure S2 — UPGMA (Unweighted Pair Group Method with Arithmetic Mean) clustering of populations according to the overall population genetic distance matrix. Each node of the dendogram represents a distinct population and is labeled regarding its continental origin (AF: Africa, OC: Oceania, EA: East Asia, CA: Central Asia, EU: Europe, AM: America). (JPG) [file pgen.1002355.s002.jpg]
