# Supplementary material for: Signatures of Environmental Genetic Adaptation Pinpoint Pathogens as the Main Selective Pressure through Human Evolution
Source: PLoS Genet. 2011 Nov 3;7(11):e1002355. doi: 10.1371/journal.pgen.1002355 (PMC3207877; doi:10.1371/journal.pgen.1002355)
Supplement: Table S4 — Enrichment of genic SNPs for different classes of similar Minor Allele Frequencies (MAF), FST or recombination rate. (PDF) [file pgen.1002355.s008.pdf]

Supplemental Table S4. Enrichment of genic SNPs for 3 different classes (low, medium and high) of similar Minor Allele Frequencies (MAF), F_{ST} or recombination rate.

	**Q^2 bin**	**Genic enrichment**		
		Low	Medium	High
**MAF**	62.5-75%	1.021	1.026	1.023
	75-87.5%	1.033	1.028	1.105
**F_{ST}**	62.5-75%	1.060	1.012	1.008
	75-87.5%	0.907	1.044	1.042
**Recombination rate**	62.5-75%	1.037	1.017	1.019
	75-87.5%	1.119	1.026	1.051
