# Supplementary material for: Signatures of Environmental Genetic Adaptation Pinpoint Pathogens as the Main Selective Pressure through Human Evolution
Source: PLoS Genet. 2011 Nov 3;7(11):e1002355. doi: 10.1371/journal.pgen.1002355 (PMC3207877; doi:10.1371/journal.pgen.1002355)
Supplement: Table S7 — Gene ontology analysis results (Function and Component domains) for SNPs mapping on genes which correlate with pathogen diversity. (PDF) [file pgen.1002355.s011.pdf]

Supplemental Table S7. Gene ontology analysis results (Function and Component domains) for SNPs mapping on genes which correlate with pathogen diversity.

Ontology term	Number of hits
**Function**	
transferase activity, transferring acyl groups	13 Hits At 9 Loci
acyltransferase activity	12 Hits At 8 Loci
transferase activity, transferring groups other than amino-acyl groups	12 Hits At 8 Loci
N-acyltransferase activity	8 Hits At 5 Loci
acetyltransferase activity	6 Hits At 4 Loci
antigen binding	5 Hits At 3 Loci
oxidoreductase activity, acting on the CH-CH group of donors	5 Hits At 4 Loci
**Component**	
endosome	7 Hits At 4 Loci
cell projection	6 Hits At 4 Loci
axoneme	5 Hits At 3 Loci
early endosome	3 Hits At 2 Loci
